# Cardiac myosin inhibitors in hypertrophic cardiomyopathy

**DOI:** 10.1186/s44348-025-00052-7

**Published:** 2025-07-07

**Authors:** Jaehyun Lim, Hyung-Kwan Kim

**Affiliations:** Division of Cardiology, Department of Internal Medicine, Seoul National University Hospital, Seoul National University College of Medicine, Seoul, Korea

**Keywords:** Cardiomyopathy, hypertrophic, Cardiac myosin inhibitor, Cardiac myosin, Therapeutics, Diastolic dysfunction, Review

## Abstract

Mavacamten, the first selective and reversible cardiac myosin inhibitor (CMI), has been introduced to the clinical arena for the treatment of obstructive hypertrophic cardiomyopathy (HCM). By reducing excessive actin-myosin cross-bridging, this agent decreases myocardial contractility and alleviates the dynamic left ventricular outflow tract (LVOT) obstruction in obstructive HCM. In the EXPLORER-HCM trial, mavacamten significantly improved exercise capacity, symptoms, and LVOT pressure gradients, while the VALOR-HCM trial proved it can obviate the need for septal reduction therapy in patients who were deemed to be candidates for septal reduction therapy. Notably, long-term data (MAVA-LTE study) has demonstrated sustained benefits up to 180 weeks, with < 10% experiencing transient reductions in left ventricular ejection fraction < 50% and only 1.3% of permanent discontinuation rate. Aficamten, a next-generation CMI with a shorter half-life, has also demonstrated comparable efficacy. Reverse remodeling following treatment was noted in both agents. In nonobstructive HCM, preliminary studies (MAVERICK-HCM trial and cohort 4 of REDWOOD-HCM trial) have reported improvements in cardiac serum biomarkers and symptoms. However, the preliminary results from phase 3 trials (ODYSSEY-HCM trial) revealed that primary endpoints were not met in nonobstructive HCM. Regarding safety, both were generally well tolerated. Although an LVEF reduction occurred in some patients, it was reversible with a dose reduction or a short-term drug cessation. These results emphasize careful dosing strategy with regular echocardiographic monitoring. Real-world data have also demonstrated consistent efficacy and safety across varying ethnic groups without new safety signals. CMI is a major advance in HCM management. However, future studies must provide data on hard clinical outcomes, such as heart failure hospitalization or death. Ongoing trials comparing CMI to traditional first-line therapies, such as β-blockers, will clarify their potential role as an initial therapeutic option.

**Supplementary Information:**

The online version contains supplementary material available at 10.1186/s44348-025-00052-7.

## Background

Hypertrophic cardiomyopathy (HCM) is a common inherited myocardial disease characterized by left ventricular (LV) hypertrophy that is unexplained by other causes and hyperdynamic systolic function with or without dynamic LV outflow tract (LVOT) obstruction. It affects roughly 1 in 200 to 500 adults​, making it the most common inherited heart disease [1–3]. Patients with HCM can present with exertional dyspnea, chest pain, or syncope, and are at increased risk for atrial fibrillation (AF), heart failure, and sudden cardiac death [4–8]. Traditional therapies—including β-blockers, nondihydropyridine calcium channel blockers, and disopyramide—alleviate symptoms by reducing heart rate or contractility, but they do not reverse myocardial hypertrophy or alter disease progression [9–12]. Severely symptomatic patients refractory to medical therapy may require septal reduction therapy (SRT) via surgical myectomy or alcohol septal ablation, both of which are invasive procedures that improve hemodynamics and quality of life, but carry procedural risk and can only be performed in a limited number of specialized HCM centers [9, 10, 13].

The development of allosteric cardiac myosin inhibitors (CMIs) represents a mechanism-specific therapeutic advance targeting the hypercontractility that is fundamental to HCM pathophysiology [14]. Mavacamten was the first CMI approved for the treatment of adults with symptomatic obstructive HCM (oHCM) in 2022, followed by ongoing trials of a second agent, aficamten [15]. CMI directly modulates cardiac sarcomere function to reduce excessive actin-myosin cross-bridge formation, thereby reducing hypercontractility and consequently LVOT pressure gradients, and improving diastolic function [16]. Initial studies demonstrated significant symptomatic and hemodynamic improvements with CMI in oHCM [17, 18]. In this review, we summarized the physiological basis, key clinical trials and real-world studies, and future perspectives of CMI in the management of HCM.

### Physiological basis of myosin inhibition in HCM

**Fig. 1:**
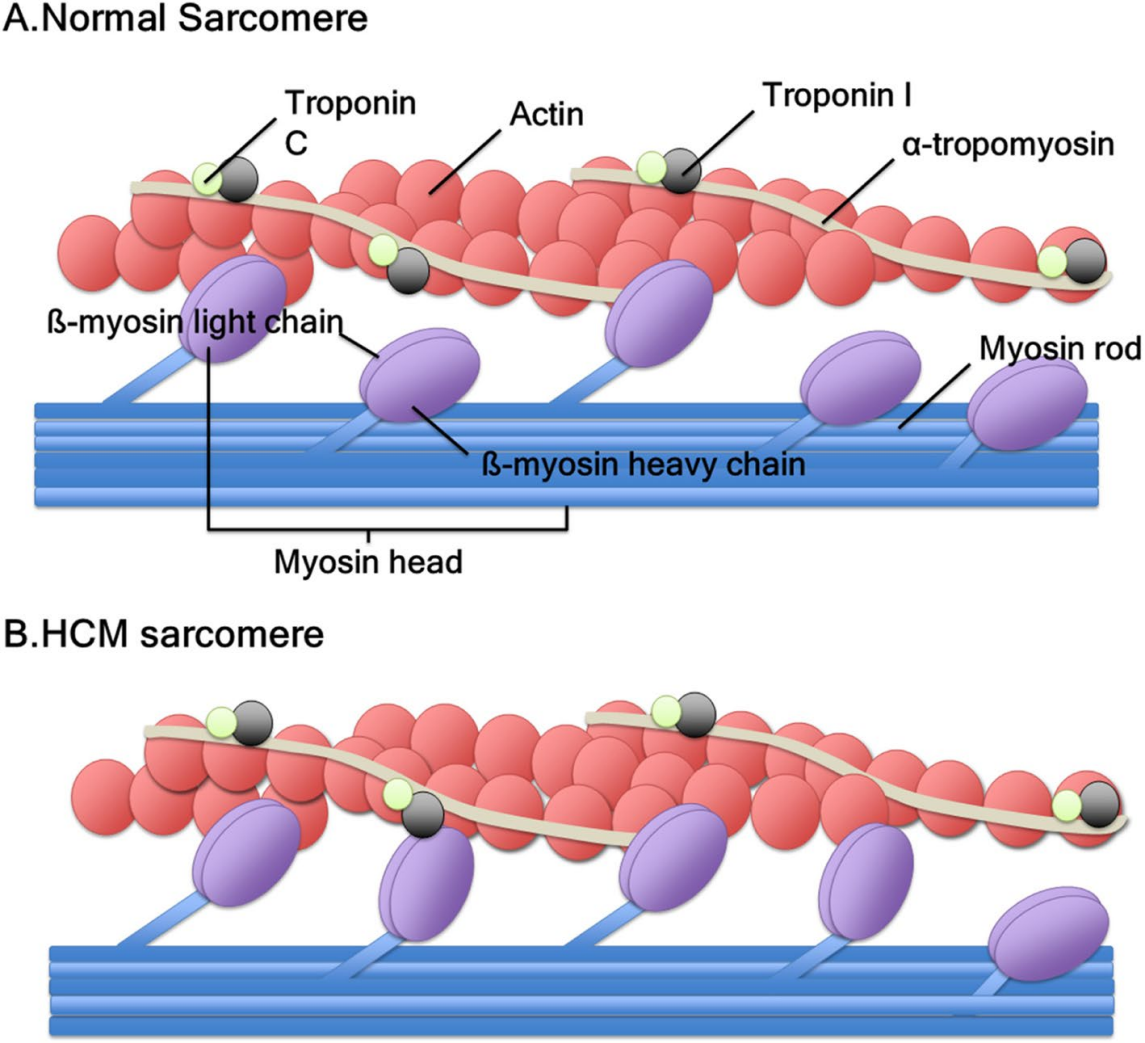
Sarcomeres of (**A**) normal heart and (**B**) hypertrophic cardiomyopathy (HCM). In a healthy heart, 40% to 50% of myosin heads are in an “off” state. Conversely, in HCM, only 15% to 20% of myosin heads are in an “off” state with increased actin-myosin cross-bridge. This state induces hypercontractility and diastolic dysfunction in the heart

HCM is caused mainly by mutations in sarcomere proteins that lead to increase myofilament contractility and impair relaxation, resulting in energy inefficiency and myocardial hypertrophy [19, 20]. The sustained hypercontractile state along with increased septal wall thickness narrows LVOT diameter, increases LVOT pressure gradients, and causes diastolic dysfunction (Fig. 1). CMIs are small molecules that bind to the myosin ATPase in cardiomyocytes and stabilize myosin heads into a state that cannot bind actin, thereby reducing the proportion of myosin cross-bridges [16]. Preclinical studies showed that through this mechanism, mavacamten, formerly MYK-461, normalizes myocardial energetics in HCM models [21], directly addressing the primary pathophysiologic abnormality in HCM [16]. Aficamten, formerly CK-274, is a next-generation CMI. In a similar, but slightly different manner, aficamten binds to an allosteric site on the myosin motor domain, thereby diminishing ATPase activity and preventing myosin from entering a force-generating state [22]. Both drugs are selective for cardiac myosin and are known to have minimal effect on smooth or skeletal muscle myosin at therapeutic concentrations [21, 22].

Despite their shared mechanism of reducing contractility, mavacamten and aficamten exhibit different pharmacokinetics: mavacamten has a longer half-life of 7 to 9 days and nonlinear pharmacokinetics, requiring 4 to 6 weeks to reach steady state [22]. Thus, careful dose titration is needed to avoid accumulation. In contrast, the half-life of aficamten is shorter: it is approximately 3.4 days, with near-steady state achieved in less than 2 weeks [22]. The dose–response relationship for aficamten is relatively shallow, resulting in smaller incremental reductions in LV ejection fraction (LVEF) per dose increase [22]. Both agents are administered orally once daily. By reducing LVOT pressure gradient and wall stress, myosin inhibition may secondarily permit reverse remodeling of hypertrophied myocardium over time, a hypothesis supported by evidence of reduced LV mass index (LVMI) and improved diastolic function on cardiac magnetic resonance or echocardiography following treatment [23, 24]. This targeted action introduces for the first time the possibility of disease-specific pharmacotherapy that not only palliates symptoms but also possibly modifies the pathophysiologic substrate.

### Clinical trials and real-world studies of CMI

In this section, results from phase 3 randomized clinical trials are presented if available; in case that phase 3 data have not yet been published, findings from phase 2 studies are summarized to provide preliminary insights. Key efficacy outcomes of these studies are summarized in Table 1 [17, 18, 25–34].

**Table 1:** Efficacy from key clinical studies

Study	Inclusion criteria	Study type	Duration (wk)	Primary endpoint	Key result
Obstructive HCM					
EXPLORER-HCM [17] (n = 251)	NYHA class II/III, LVEF ≥ 55%, any LVOT PG ≥ 50 mmHg	RCT	30	Increased pVO_2_ 1.5 mL/kg/min and ≥ 1 NYHA class improvement or increase pVO_2_ 3.0 mL/kg/min and stable NYHA class	37% vs. 17% met primary LVOT PG, − 47 mmHg vs. − 10 mmHg Increased pVO_2_, 1.4 mL/kg/min vs. –0.1 mL/kg/min ≥ 1 NYHA class improvement, 65% vs. 31%
MAVA-LTE [25] (n = 211)	EXPLORER-HCM completers after 8-wk washout	Prospective cohort	Median, 166 Maximum, 252	Long-term safety and efficacy	Resting LVOT PG, − 40 mmHg Valsalva LVOT PG, − 55 mmHg ≥ 1 NYHA class improvement, 78% NT-proBNP level at week 180, − 562 ng/L (IQR, − 1,162.5 to − 209 ng/L)
VALOR-HCM [18] (n = 112)	NYHA class III/IV or NYHA II with syncope, SRT candidates, LVEF ≥ 60%, any LVOT PG ≥ 50 mmHg	RCT	16	Composite of proceeding to or remaining eligible for SRT	18% vs. 77% met primary Postexercise LVOT PG, − 37 mmHg ≥ 1 NYHA class improvement, 63% vs. 21% NT-proBNP level, − 399 ng/L vs. 40 ng/L Troponin I level, − 9.2 ng/L vs. 0.07 ng/L
Week 32 [26] (n = 108)	VALOR-HCM completers, placebo group crossed over to mavacamten	Prospective cohort	32	Same as VALOR-HCM	10.7% (original mavacamten) vs. 13.5% (cross over to mavacamten from placebo) met primary Resting LVOT PG, –33 mmHg Valsalva LVOT PG, − 43 to − 53 mmHg ≥ 1 NYHA class improvement, 90.6% vs. 70%
Week 56 [27] (n = 108)	Same as VALOR-HCM week 32	Prospective cohort	56	Same as VALOR-HCM	8.9% vs. 19.2% met primary Resting LVOT PG, –33 mmHg vs. –37 mmHg Valsalva LVOT PG, − 46 mmHg vs. − 55 mmHg ≥ 1 NYHA class improvement, 90.6% vs. 70%
Week 128 [28] (n = 108)	Same as VALOR-HCM week 32	Prospective cohort	128	Same as VALOR-HCM	14.3% vs. 17.3% met primary Resting LVOT PG, − 34 mmHg vs. − 35 mmHg Valsalva LVOT PG, − 53 mmHg vs. − 67 mmHg ≥ 1 NYHA class improvement, 86% vs. 75%
EXPLORER-CN [29] (n = 81)	Same as EXPLORER-HCM	RCT	30	Reduced Valsalva LVOT PG	Valsalva LVOT PG, − 51 mmHg vs. + 19 mmHg ≥ 1 NYHA class improvement, 59% vs. 15 NT-proBNP reduction, 82% greater LVMI by CMR, − 26 g/m^2^ vs. 4 g/m^2^ LAVI by CMR, − 17 mL/m^2^ vs. 1 mL/m^2^ Max wall thickness, − 3.0 mm vs. 0.5 mm
SEQUOIA-HCM [33] (n = 282)	NYHA class II/III, LVEF ≥ 60%, resting LVOT PG ≥ 30 mmHg and Valsalva LVOT PG ≥ 50 mmHg	RCT	24	pVO_2_	1.8 vs. 0 mL/kg/min; KCCQ-CSS improvement ≥ 1 NYHA class improvement, 59% vs. 24% Valsalva LVOT PG, − 48 mmHg vs. + 2 mmHg
Korean cohort [30] (n = 46)	NYHA class ≥ II, LVEF ≥ 55%, any LVOT PG ≥ 30 mmHg	Prospective cohort	Median, 21	Efficacy and safety	Resting LVOT PG, − 40 mmHg Valsalva LVOT PG, − 56 mmHg ≥ 1 NYHA class improvement, 58%
Japanese cohort [31] (n = 38)	NYHA class II/III, LVEF ≥ 60%, any LVOT PG ≥ 50 mmHg, Valsalva LVOT PG ≥ 30 mmHg	Prospective cohort	30	Efficacy and safety	Resting LVOT PG, − 61 mmHg Valsalva LVOT PG, − 71 mmHg Postexercise LVOT PG, − 61 mmHg
Nonobstructive HCM					
MAVERICK-HCM [32] (n = 59)	NYHA class II/III	RCT	16	Safety (LVEF, adverse events)	No significant difference in improvement of e’, E/e’, pVO_2_, NYHA class
REDWOOD-HCM cohort 4 [34] (n = 40)	NYHA II/III, LVEF ≥ 60%, NT-proBNP > 300 pg/mL	Prospective cohort	10	Safety (LVEF < 50%)	≥ 1 NYHA class improvement, 55% Increased KCCQ-CSS NT-proBNP level decreased, 56% Troponin I level decreased, 22%

HCM, hypertrophic cardiomyopathy; IQR, interquartile range; KCCQ-CSS, Kansas City Cardiomyopathy Questionnaire–Clinical Summary Score; LAVI, left atrial volume index; LVMI, left ventricular mass index; LVEF, left ventricular ejection fraction; LVOT, left ventricular outflow tract; NT-proBNP, N-terminal pro–B-type natriuretic peptide; NYHA, New York Heart Association; PG, pressure gradient; pVO_2_, peak oxygen consumption; RCT, randomized controlled trial; SRT, septal reduction therapy

### Mavacamten trials

#### EXPLORER-HCM trial

The pivotal phase 3 EXPLORER-HCM trial evaluated mavacamten in adults with symptomatic oHCM (New York Heart Association [NYHA] class II/III) and resting or provoked LVOT pressure gradient of ≥ 50 mmHg despite maximal medical therapy [17]. In this multicenter, double-blind trial, 251 patients were randomized to either mavacamten or placebo for 30 weeks. Mavacamten was started at 5 mg daily with dose adjustment at 8 and 14 weeks to achieve target LVOT pressure gradient of < 30 mmHg and a mavacamten plasma concentration between 350 and 700 ng/mL. The primary endpoint, a composite of improved exercise capacity and symptoms, was achieved in 37% of the mavacamten versus 17% of placebo group (*P* = 0.0005). Specifically, mavacamten-treated patients had a greater likelihood of achieving the primary endpoint, i.e., ≥ 1.5 mL/kg/min increase in peak oxygen consumption (pVO_2_) and ≥ 1 NYHA class improvement or ≥ 3.0 mL/kg/min pVO_2_ increase with no worsening in NYHA class. Overall, pVO_2_ increased by a mean + 1.4 mL/kg/min on mavacamten versus a slight decrease on placebo (*P* = 0.0006), and the postexercise LVOT pressure gradient decreased by 47 mmHg in the mavacamten versus 10 mmHg in the placebo arm (*P* < 0.0001). Symptomatically, 65% of patients on mavacamten improved by ≥ 1 NYHA class versus 31% with placebo, and quality-of-life scores (Kansas City Cardiomyopathy Questionnaire–Clinical Symptom Score [KCCQ-CSS]) improved by 13.6 points, which was 9.1 points higher than placebo (*P* < 0.0001). By week 30, 27% in the mavacamten arm achieved complete treatment response, i.e., achieving LVOT pressure gradients < 30 mmHg plus NYHA class I, compared to only 1% in the placebo arm.

Importantly, the substudies of the EXPLORER-HCM trial demonstrated reverse remodeling of cardiac structure and function: one study that utilized cardiac magnetic resonance demonstrated reductions in absolute intracellular myocardial mass index, LVMI, maximum LV wall thickness, and left atrial volume index (LAVI) [23]. Another substudy of EXPLORER-HCM with echocardiography showed additional benefits of mavacamten in improvements of mitral valve systolic anterior motion and diastolic function [24].

#### MAVA-LTE study

The longest follow-up study to date of mavacamten therapy in oHCM, the 5-year MAVA-LTE study, has demonstrated sustained improvements in key clinical and hemodynamic parameters [25]. Among 231 patients enrolled, long-term treatment with mavacamten (median duration 166 weeks) led to persistent reductions in resting (–40.3 ± 32.7 mmHg) and post–Valsalva maneuver LVOT pressure gradients (–55.3 ± 33.7 mmHg), alongside marked improvements in NYHA functional class, N-terminal pro–B-type natriuretic peptide (NT-proBNP) levels, and patient-reported outcomes including Hypertrophic Cardiomyopathy Symptom Questionnaire shortness of breath domain score and EQ-5D-5L scores​. The majority of patients (91.3%) from the EXPLORER-HCM trial remained on treatment. Excessive baseline hypercontractility was normalized, with mean central-read LVEF decreasing from 73.9% at baseline to 63.9% at week 180, and over 739 patient-years of exposure, only 20 individuals (8.7%) experienced transient, reversible reductions in LVEF < 50%. Although findings from the MAVA-LTE study reinforced the long-term safety and durable symptomatic and structural benefits of myosin inhibition, its safety in the longer term remains to be validated.

#### VALOR-HCM trial

To address whether mavacamten could obviate the need for invasive therapy in advanced oHCM, the phase 3 VALOR-HCM trial investigated 112 patients with severe symptoms (NYHA class III/IV) who met the guideline criteria for SRTs [18]. Participants were randomized to mavacamten or placebo for 16 weeks. The primary endpoint was a composite of proceeding to SRT or remaining eligible for SRT per 2011 American College of Cardiology/American Heart Association guideline at week 16. In this study, mavacamten markedly reduced the need for SRT: only 18% of patients on mavacamten still met the SRT criteria or chose to undergo SRT, compared to 77% in the placebo arm (*P* < 0.0001) [18]. Moreover, all secondary endpoints favored mavacamten, including greater improvements in ≥ 1 NYHA class (63% vs. 21%, *P* < 0.001), resting LVOT pressure gradient (–36.0 ± 28.8 vs. –1.5 ± 26.5 mmHg), and NT-proBNP and troponin I levels. No patients on mavacamten experienced serious adverse events, including death, myocardial infarction, or ventricular tachyarrhythmias. This landmark study established CMI as a viable alternative to invasive SRT in advanced, highly symptomatic oHCM, allowing many patients to avert surgery by achieving sufficient symptomatic improvement with mavacamten. It also demonstrated that even very symptomatic, patients with extremely high LVOT pressure gradients can respond robustly to mavacamten.

Extended follow-up of VALOR-HCM participants has also provided similar insights into long-term outcomes. After the 16-week randomized phase, all patients were offered open-label mavacamten. In this extended period, both the original mavacamten and cross-over groups (those whose therapy transitioned from placebo to mavacamten) demonstrated durable benefit: only 11% to 14% of patients in the entire cohort remained eligible for SRT at week 32 [26]. One- and two-year results through 56 and 128 weeks of mavacamten therapy were subsequently reported [27, 28]. In the most recently published 128-week analysis, the benefits observed at week 16 were sustained. In this latest analysis, 108 patients were followed up for 128 weeks of the study among the 112 initial study participants [18]. Of note, four patients excluded from the analyses included two patients who underwent SRT before week 16, and two patients who discontinued the study. Notably, nearly 90% of patients remained free of SRT through 2.5 years of mavacamten therapy. More specifically, 17 of 108 (15.7%) were either indicated for SRT or could not be definitively evaluated due to incomplete data [28]. The vast majority maintained NYHA class I/II status with low LVOT pressure gradients, with only 2.8% remaining in NYHA III/IV, although missingness in NYHA class existed in 11.1%. Clinical metrics like resting and provoked LVOT pressure gradients, KCCQ score, and NT-proBNP and troponin I levels were also significantly improved in both groups at week 128. Furthermore, echocardiographic indices including LVMI, LAVI, septal E/e’ and interventricular septal thickness were significantly decreased. It should be noted that long-term mavacamten was well tolerated with a low incidence of LVEF reduction to less than 50%: during follow-up from baseline to week 128, 15 (13.9%) experienced LVEF < 50%, however, only 3 required permanent discontinuation due to prespecified criteria of LVEF < 30% or < 50% with the lowest dose. Other 12 patients, who experienced LVEF < 50% before week 56, could safely restart mavacamten with a lower dose, and remained asymptomatic with preserved LVEF throughout the rest of the follow-up period to week 128.

#### EXPLORER-CN trial

Given the limited representation of Asian populations in previous trials, EXPLORER-CN trial, a phase 3 randomized, double-blind, placebo-controlled study, was conducted across 12 centers in China [29]. This is of value as Asian HCM patients exhibit distinct characteristics compared to Western patients, including less frequent obstructive physiology, more frequent apical hypertrophy, lower baseline LVEF, and higher prevalence of CYP2C19 poor metabolizer genotypes [35–38]. The inclusion criteria of the EXPLORER-CN trial were identical to that of EXPLORER-HCM [17]. Mavacamten was started at a lower starting dose of 2.5 mg once daily and titrated similar to the protocol used in the previous EXPLORER-HCM trial [17]. Among 81 patients randomized 2:1 to mavacamten or placebo for 30 weeks, mavacamten significantly reduced LVOT pressure gradient with Valsalva maneuver (least-squares mean difference, –70.3 mmHg; P < 0.001), with substantial improvements in resting gradient, NYHA class, KCCQ-CSS score, cardiac biomarkers (NT-proBNP and troponin I levels), and LVMI​. Notably, 48.1% of patients achieved Valsalva gradient < 30 mmHg, and 59.3% had NYHA class improvement, both markedly superior to placebo. Importantly, no patients showed decreased LVEF that went below 50%. Although it was not clearly reported in the publication, dose of mavacamten prescribed at week 30 was 5 mg in 59.3% and 10 mg in 29.6% of patients enrolled, which was not so different from dose reported in a Risk Evaluation and Mitigation Strategy (REMS) Program (described below).

#### REMS Program

To ensure post-approval safety oversight (i.e., to reduce the risk of systolic heart failure), the US Food and Drug Administration (FDA) mandated a REMS Program for mavacamten, focusing on monitoring LV systolic function and potential drug interactions. A recent report from the REMS Program, which includes over 6,000 patients treated between April 2022 and February 2024, provides the largest real-world dataset on mavacamten to date​ [39].

Among 6,299 patients who received at least one dose of mavacamten, 5,573 patients submitted patient status form and were analyzed. At its 6-month analysis, 70% reported LVOT pressure gradient with Valsalva maneuver of < 30 mmHg. Notably, 256 (4.6%) experienced a reduction in LVEF to < 50%, and 71 (1.3%) required hospitalization for heart failure. These rates are similar to or little lower than that observed in previous trials. Among 1,929 patients with over 1-year use of mavacamten, similar proportion (*n* = 78, 4.0%) of patients experienced LVEF reduction to < 50%, 29 patients (1.5%) experienced heart failure hospitalization, and 4 patients (0.2%) experienced LVEF < 50% and heart failure hospitalization, all of whom resumed later treatment at a lower dose. As a whole, mavacamten was safe and well tolerated in a real-world environment and thus regular monitoring schedule after 3-month of mavacamten initiation has recently been modified from 3- to 6-month interval in the United States.

#### Korean observational study

**Fig. 2:**
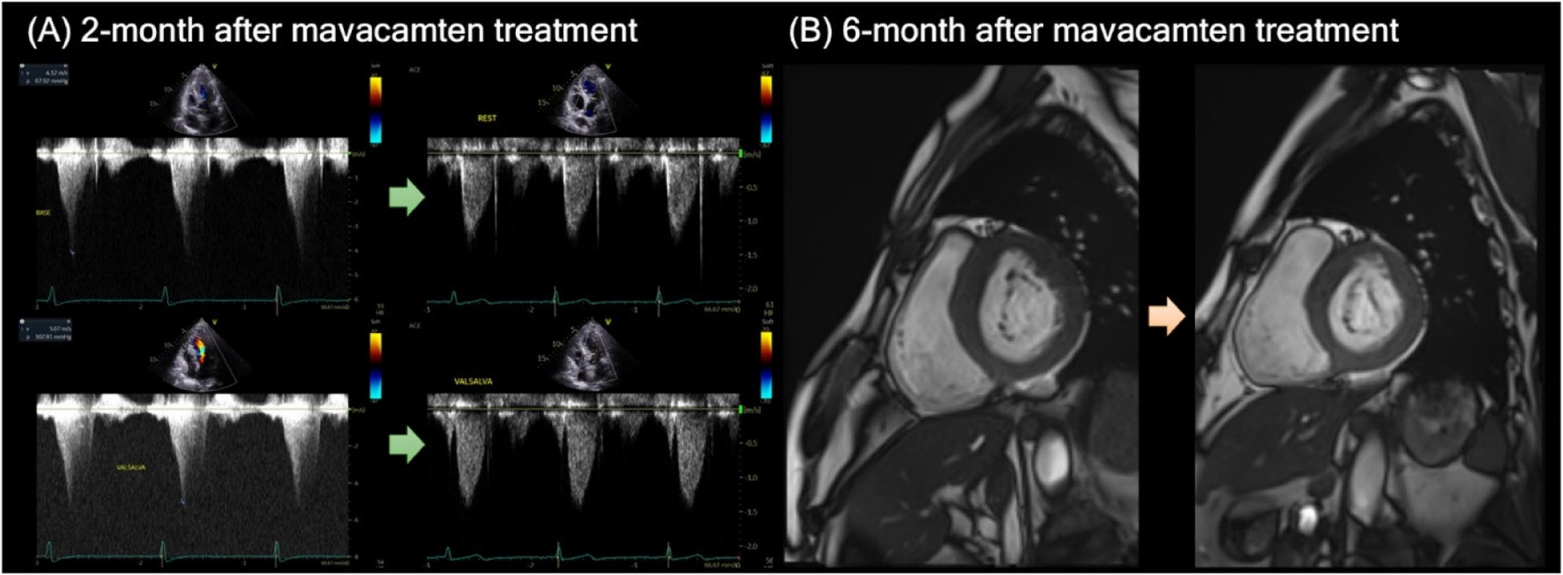
A real-world example of 54-year-old man who showed complete response after 2-month of mavacamten treatment. **A** Resting left ventricular outflow tract pressure gradient decreased from 67 mmHg to a negligible level, and that with Valsalva maneuver also decreased from 103 mmHg to a similarly low level. Correspondingly, his symptoms improved from New York Heart Association class II to class I. **B** Cardiac magnetic resonance revealed that his septal wall thickness significantly decreased from 17 to 15 mm after 6 months of mavacamten therapy

A recent report from seven university hospitals in Korea has demonstrated similar efficacy and safety of mavacamten in Asian patients [30]. In this prospective, observational, multicenter study of 46 patients, mean reduction in LVEF was − 3.4% and 1 patient (2.2%) experienced LVEF to < 50% during a median follow-up duration of 147 days. Notably, significant reductions of − 40 and − 68 mmHg in LVOT pressure gradient during resting and with Valsalva maneuver, respectively, were observed (Fig. 2A, Supplementary Videos).

This study reported that reductions in NT-proBNP levels was strongly correlated with LVOT pressure gradient reductions with conditional R^2^ value of 0.926. Although high interpatient variability existed, this study suggests that NT-proBNP may serve as a marker for monitoring therapeutic response at a single patient level and partially replace the need for frequent echocardiographic assessments mandated by the REMS Program, which, while ensuring safety, may impose excessive cost and echocardiographic laboratory burden in a real-world practice​. Of note, even a short-term treatment of mavacamten was accompanied by reduction in maximal LV wall thickness of − 1.5 mm (*P* = 0.018) and LAVI of − 5.1 mL/m^2^ (*P* = 0.315), although the latter did not reach statistical significance (Fig. 2B). Nonetheless, these findings suggest positive effect of LV reverse remodeling induced by mavacamten treatment.

#### Japanese observational study

Another prospective observational multicenter study in Asia was conducted in Japan with 38 patients with oHCM [31]. After 30-week treatment of mavacamten, resting, Valsalva, and postexercise LVOT pressure gradient showed significant decrease of − 61, − 71, and − 61 mmHg, respectively. Cardiac biomarkers of NT-proBNP and troponin I levels also showed significant decrease, with the geometric mean ratios of baseline to at week 30 showing 0.18 and 0.32, respectively.

**Fig. 3:**
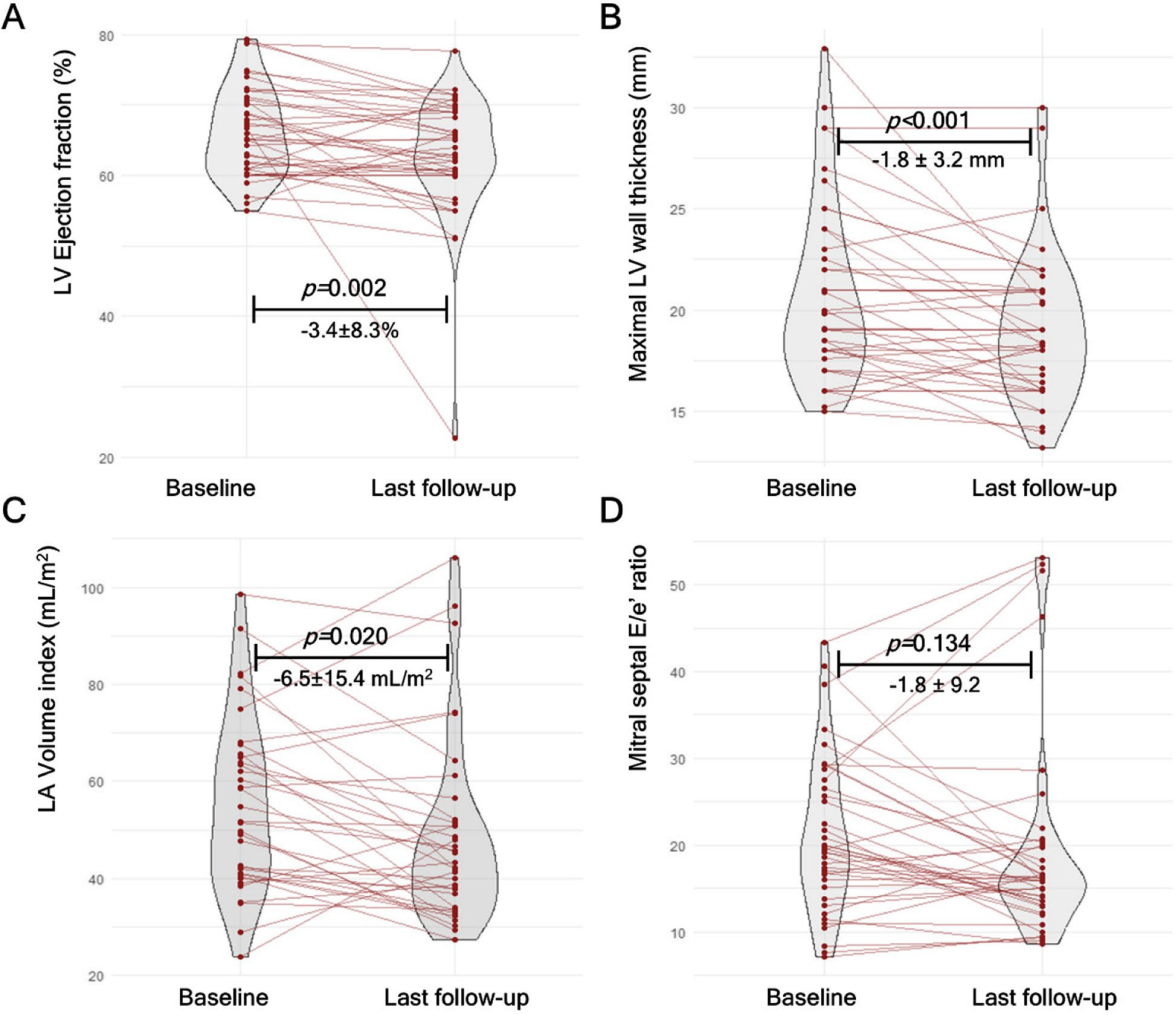
Data from Korean prospective multicenter real-world study demonstrating cardiac reverse remodeling after a median mavacamten treatment of 21 weeks. Violin plots showing (**A**) changes in left ventricular (LV) ejection fraction, (**B**) changes in maximal LV wall thickness, (**C**) changes in left atrial (LA) volume index, and (**D**) changes in mitral septal E/e’ ratio

Importantly, no new safety concerns were identified in this study. Furthermore, although the number of study participants was small, this study suggested that echocardiography-based dose modification scheme well-guided appropriate use of mavacamten dose across CYP2C19 metabolizer phenotypes, and accordingly resulted in similar rates of adverse events (Fig. 3).

#### MAVERICK-HCM for nHCM

Although patients with nonobstructive HCM (nHCM) also suffer exercise intolerance and diastolic dysfunction, pharmacologic options tackling pathophysiology lack beyond symptom control. The phase 2 MAVERICK-HCM trial investigated mavacamten in symptomatic nHCM [32]. In this dose-ranging study, 59 patients were randomized to mavacamten (target mean concentration, 200 or 500 ng/mL) or placebo for 16 weeks. The primary focus was safety, given concerns about reducing contractility. Mavacamten was generally well tolerated in this cohort, with no excess occurrence of serious adverse events compared to placebo. As expected, the mean LVEF declined slightly in mavacamten group compared to placebo (–4.1% ± 8.0% vs. –2.3% ± 4.9%), and 12.5% of participants with mavacamten were reported to have LVEF ≤ 45% at weeks 11 to 12. However, all patients recovered successfully after drug cessation.

Although the trial was not powered for efficacy, exploratory endpoints suggested potential benefits: mavacamten-treated patients had significant reductions in NT-proBNP (*P* = 0.0005) and troponin I levels (*P* = 0.009) and modest improvements in NYHA class was shown, whereas KCCQ scores and pVO_2_ did not demonstrate significant difference versus placebo. Although a prespecified composite functional endpoint mirroring that of EXPLORER-HCM was reached in 22.5% of mavacamten-treated versus 21.1% of placebo patients and did not significantly differ, subgroup analyses indicated that patients with higher baseline troponin I or filling pressures might derive greater symptomatic benefit from mavacamten. More recently, however, preliminary results from the phase 3 ODYSSEY-HCM trial reported that mavacamten did not achieve the primary endpoint [40]. Although taking a closer look at the data is required following the formal publication of the main results, whether nHCM is a distinct disease entity from oHCM, or whether the presence of specific subgroups may derive benefit from mavacamten as suggested in phase 2 studies, remains an important area for a future investigation.

### Aficamten trials

#### SEQUOIA-HCM

The phase 3 SEQUOIA-HCM trial tested aficamten in patients with NYHA II or III oHCM [33]. In this recently published trial, 282 patients with symptomatic oHCM were randomized 1:1 to aficamten or placebo on top of standard therapy for 24 weeks. Aficamten was initiated at 5 mg daily with possible dose increase by 5-mg increments up to 20 mg daily at weeks 2, 4, and 6 based on echocardiographic guidance. The primary endpoint was change in exercise capacity, measured by pVO_2_ on cardiopulmonary exercise testing.

The SEQUOIA trial met its primary endpoint: at week 24, patients on aficamten demonstrated + 1.7 mL/kg/min greater increase in pVO_2_ than placebo (*P* < 0.001). This corresponded to a mean improvement of + 1.8 mL/kg/min from baseline in the aficamten arm, versus essentially no change in the placebo arm. All key secondary endpoints also favored aficamten. By week 24, aficamten-treated patients had profound reductions in LVOT pressure gradient with Valsalva maneuver (e.g., − 50 mmHg more than placebo, *P* < 0.001) and 58.5% of the aficamten group improved by at least one NYHA class, compared with 24.3% on placebo. Furthermore, improvements in KCCQ-CSS scores, total workloads during cardiopulmonary testing, and NT-proBNP level were significantly better with aficamten.

The safety profile of aficamten in SEQUOIA-HCM was favorable and generally similar to placebo. The incidence of LVEF dropping below 50% was low (3.5% on aficamten vs. 0.7% on placebo) and none of these transient LVEF reductions led to heart failure hospitalizations. All patients with LVEF reduction were managed with protocol-directed dose adjustments and no patients on aficamten required permanent discontinuation due to systolic dysfunction. AF occurred at a similarly low rate in both groups of 2% to 3%.

#### REDWOOD-HCM cohort 4 for nHCM

Given positive signals from MAVERICK-HCM with mavacamten [32], aficamten has also been evaluated in nHCM patients [34]. An open-label phase 2 study with cohort 4 of REDWOOD-HCM enrolled 41 patients with NYHA class II/III, nHCM patients to receive aficamten for 10 weeks. The goal was to assess safety and efficacy. The dose of aficamten was titrated according to echocardiographic LVEF guidance, aiming for a target an LVEF of 50% to 54%.

By week 24, patients demonstrated improvements in exercise tolerance and functional status: 55% improved at least one NYHA class and 56% experienced clinically meaningful gains in KCCQ-CSS (≥ 5-point increase), with 26% reporting large (≥ 20-point increase) improvements. Cardiac biomarkers also significantly declined: NT-proBNP level fell by 56% (*P* < 0.001) and troponin I level by 22% (*P* < 0.005). In addition, the study demonstrated improvement in diastolic parameters of septal and lateral e′ velocities. Mean LVEF modestly declined (–5%), with three patients experiencing asymptomatic LVEF < 50%, all recovering after washout. One sudden cardiac death occurred in a high-risk patient with prior aborted sudden cardiac death and an implantable cardioverter-defibrillator (ICD) in situ, deemed unrelated to the study drug. Further evidence on aficamten in nHCM is anticipated from the ongoing ACACIA-HCM trial (ClinicalTrials.gov identifier: NCT06081894) that investigates aficamten effects on subjective and objective improvement in symptom and exercise performance.

## Safety and adverse effects of CMIs

Key safety outcomes of studies using CMIs are summarized in Table 2 [17, 18, 25–34, 39].

**Table 2:** Safety outcomes in key clinical studies

Study	LVEF < 50%	ΔLVEF	Permanent discontinuation	Other serious adverse events
Obstructive HCM				
EXPLORER-HCM [17]	6% (transient, recovered)	− 3.9% (week 30)	NA	None excess vs. placebo, 8% vs. 9%
MAVA-LTE [25]	8.7% (transient, recovered)	− 9.6% (week 156) − 11.0% (week 180)	1.3% (n = 3)	No new safety signals over 739 patient-years
VALOR-HCM [18]	3.6% (transient, recovered)	− 3.4% (week 16)	0%	None reported (no deaths, AMIs, or VT/VF)
Week 32 [26]	12.5% (original mavacamten) vs. 3.8% (cross-over group; transient, recovered)	− 2.9% to − 4.7% (week 32)	1.9% (n = 1) in cross-over group had LVEF ≤ 30% with AF and HF LVEF recovered	AF (n = 3), CHF (n = 1), PTE (n = 1), ARF (n = 1), COVID-19 (n = 1), CDI (n = 1), fall (n = 1), nephrolithiasis (n = 1), peripheral venous disease (n = 1)
Week 56 [27]	11.1% (n = 12; reinitiated, n = 9)	− 4.0% (week 56)	2.8% (n = 3): recovered, n = 2; sudden death, n = 1	Week 32 events + ventricular arrhythmia (n = 1), severe GERD (n = 1), drug administration site reaction (n = 2), life-threatening syncope (n = 1)
Week 128 [28]	13.9% (n = 15; reinitiated, n = 12)	NA	No additional permanent discontinuation between week 56–128	Week 56 events + AF (n = 7), AMI (n = 1), HHF (n = 1), stroke (n = 1), cardiac death (n = 1), appropriate defibrillation (n = 1), ventricular arrhythmia (n = 1)
EXPLORER-CN [29]	0%	+ 3.7% (week 30)	0%	Only occurred in mavacamten group: AF/AFL (5.7%), sinus arrest (1.9%), sinus node dysfunction (1.9%), hypotension (1.9%), hemorrhoids (1.9%), ankle fracture (1.9%)
REMS Program [39]	4.6% (transient, recovered)	NA	0%	NA
Korean cohort [30]	2.2% (transient, recovered)	− 3.4% (median, 21 weeks)	30%^a)^	Deaths (4.3%), AF (4.3%), stroke (4.3%), HHF (2.2%)
Japanese cohort [31]	2.8% (transient, recovered)	− 2.9% (week 30)	0%	AF (7.9%), palpitation (5.3%)
SEQUOIA-HCM [33]	3.5% (transient, recovered)	− 4.8% (week 24)	0%	AF (2.8% vs. 2.9%), VF (0% vs. 0.7%), palpitation (7.0% vs. 2.9%), hypertension (7.7% vs. 2.1%) Any serious adverse events, 5.6% vs. 9.3%
Nonobstructive HCM				
MAVERICK-HCM [32]	12.5% (LVEF ≤ 45%, recovered)	− 4.1% (week 16)	0%	AF (5.1%), arthritis (2.6%), renal failure (2.6%) Serious adverse events, 10% vs. 21%
REDWOOD-HCM cohort 4 [34]	8% (transient, recovered)	− 5.4% (week 10)	0%	Sudden cardiac death (n = 1, preexisting high-risk patient, unrelated)

ARF, acute respiratory failure; AF, atrial fibrillation; AFL, atrial flutter; AMI, acute myocardial infarction; CDI, *Clostridium difficile* infection; CHF, congestive heart failure; GERD, gastroesophageal reflux disease; HCM, hypertrophic cardiomyopathy; HF, heart failure; HHF, hospitalization for heart failure; LVEF, left ventricular ejection fraction; NA, not available; PTE, pulmonary thromboembolism; REMS, Risk Evaluation and Mitigation Strategy; VF, ventricular fibrillation; VT, ventricular tachycardia

^a)^Main reason for discontinuation was financial burden (20% of total population)

### Decline in systolic function

The principal safety concern with CMIs is excessive reduction of systolic function leading to heart failure hospitalization. Both mavacamten and aficamten can lower LVEF in a dose-dependent fashion. In clinical trials, including phase 2 and 3 studies, around 10% of patients experienced transient LVEF < 50% [28, 41]. However, in the pivotal trials, no patients developed irreversible heart failure when appropriate dose adjustments or drug discontinuation were implemented. The systolic function depression is typically reversible with drug interruption considering the agents’ pharmacodynamics [21, 22]. Real-world postmarketing data on mavacamten have thus far aligned with trial safety, showing that significant LV dysfunction is uncommon when adhering to recommended monitoring and dosing [39]. Nevertheless, it should be emphasized that in the longest study to date, MAVA-LTE study, patients receiving mavacamten experienced a mean LVEF reduction of − 11.0% at week 180, although the final LVEF was still above 60%. This reduction is substantially greater than those observed in shorter-term studies (Table 2) [17, 18, 25–34, 39] and may exceed the anticipated decline in LVEF with CMIs [25]. However, this decline can be considered normalization of LV hypercontractility, since the LVEF of the patients fell into the “ideal” normal value from 73.9% at baseline to 63.9% at week 180. Moreover, significant decline in LVEF leading to permanent discontinuation occurred only in 1.3% and further reduction in LVEF was not observed after week 152 according to VALOR-HCM extension study [28].

### Arrhythmias

HCM patients are predisposed to AF and ventricular arrhythmias from myocardial scarring [42]. It is reassuring that CMI therapy has not been associated with proarrhythmic effects. In the EXPLORER-HCM and SEQUOIA-HCM trials, the two pivotal clinical trials of mavacamten and aficamten, the incidence of AF was low and similar between treatment and placebo arms: 2% (mavacamten) versus 3% (placebo) in the EXPLORER-HCM [17] and 2.8% (aficamten) versus 2.9% (placebo) in the SEQUIA-HCM trial [33] (Table 2). The longest follow-up study to date, the MAVA-LTE cohort, provides important insights in long-term arrhythmic risks [25]. In this study, AF incidence was 14.3%, and excluding patients with previous AF results in 7.8% of de novo AF incidence [25]. This incidence is lower than that shown in observational study of 18% [43]. Moreover, in the MAVA-LTE study, the exposure-adjusted rate of AF declined over time—from 6.57 cases per 100 patient-years during the initial 60 weeks to 4.50 per 100 patient-years by week 252 [43]. This trend suggests that sustained treatment with mavacamten does not increase AF risk and may, in fact, contribute to lowering AF incidence.

In studies to date, ventricular tachyarrhythmias were rare, and no increase in nonsustained ventricular tachycardia or ICD shocks has been reported on therapy relative to baseline or placebo. However, further long-term data on ventricular arrhythmic events are required. 

### Other adverse effects

Across trials, systemic side effects have been insignificant. Unlike β-blockers, CMIs have no direct effect on blood pressure or central nervous system. Also, side effects like fatigue, hypotension, or bradycardia have shown similar rates to placebo. There have been no drug-specific organ toxicities identified. Elevations in liver enzymes or other laboratory abnormalities have not occurred at rates above placebo. However, since both drugs are metabolized hepatically—mavacamten primarily by CYP2C19 and CYP3A4, aficamten by CYP3A4—clinicians must be mindful of drug-drug interactions that could possibly raise plasma levels of CMIs, as increases in plasma level of mavacamten would cause acute heart failure. For mavacamten, for example, caution is warranted in concomitant use of strong CYP2C19 or CYP3A4 inhibitors, such as certain azoles, fluvoxamine, verapamil, or omeprazole/esomeprazole, as mavacamten concentration is likely to increase [44]. On the contrary, concomitant use of midazolam or rifampin would mitigate the effect of mavacamten [44]. Concomitant use of oral contraceptives may result in unwanted pregnancy [44]. Furthermore, animal studies of mavacamten showed embryo-fetal toxicities, such as cardiac malformations [9].

Aficamten’s metabolism may be similarly affected, though definitive interaction data are pending [9]. Data currently available suggest that aficamten possesses less drug-drug interactions [45]. Nonetheless, patients should be counseled to alert providers of new medications, and aficamten would likely have similar precautions regarding pregnancy.

### Monitoring

In order to balance symptom relief and LVOT pressure gradient reduction with preservation of adequate systolic function, therapy with CMIs requires ongoing clinical and echocardiographic monitoring [9]. Current recommendations for mavacamten include echocardiography every 4 weeks during dose titration for the first 3 months and every 3 months thereafter to assess LVOT pressure gradient and LVEF [9]. If LVEF drops to < 50%, the drug should be temporarily discontinued and reassessed. This enhanced need for regular imaging follow-up is indeed a hurdle in widespread adoption of the medication. Laboratory monitoring of NT-proBNP may substitute in some part of echocardiography, as it has shown significant correlation with decrease in LVOT pressure gradient [30]. Meanwhile, it also remains to be validated whether aficamten’s more rapid equilibrium and shorter effective half-life might allow a less intensive monitoring schedule. Until more experience and data are available, similar vigilance is warranted when prescribing aficamten. Recent real-world data from the REMS Program showed that mavacamten was safe and well tolerated and thus FDA has recently accepted for prolongation of regular monitoring schedule from 3 to 6 months after 3 months of mavacamten initiation.

## Integration into clinical practice and future perspectives

Contemporary HCM guidelines have currently incorporated mavacamten as a treatment option for symptomatic oHCM. The 2024 American guidelines assign a class I recommendation for use of mavacamten in symptomatic patients with oHCM despite use of maximal first-line therapy [9]. Notably, mavacamten has been positioned in the same hierarchy with SRT. The 2023 European Society of Cardiology cardiomyopathy guidelines have also recognized mavacamten as an effective therapy for symptomatic oHCM [10]. Indeed, European guidelines suggest prioritized use of mavacamten over SRT, favoring a stepwise approach starting with reversible pharmacologic intervention before definitive invasive SRT.

Nonetheless, some patients would still prefer a one-time procedural “fix” over lifelong medication, whereas others favor avoiding invasive treatments and its risks [9, 10]. Therefore, shared decision-making is a key in choosing between long-term CMI treatment versus invasive SRT for those who are eligible for both. The nonresponders to CMIs should also be emphasized. For example, in the MAVA-LTE cohort, approximately 17% of patients did not achieve LVOT pressure gradient with Valsalva maneuver of < 30 mmHg [25]. Similarly, in the VALOR-HCM that enrolled surgical candidates of symptomatic oHCM patients, approximately one in seven patients still remained surgical candidates or underwent SRT at week 128, although more than half of reasons for meeting the primary endpoints (being a surgical candidate or undergoing SRT) were SRT-unevaluable status [28]. In these patients, SRT would still be valid and remains as the only therapeutic option.

In contrast, patients who achieve good initial response must stay on therapy to maintain benefits; symptom relapse can occur after drug cessation, as underlying hypercontractility and dynamic LVOT obstruction come back after drug discontinuation. Thus, commitment to long-term therapy is crucial in chronic CMI users. In this scenario, cost and insurance coverage impact its use: CMI is a high-cost medication, and ensuring coverage for both the drug and required monitoring is a crucial part of practical management. The Korean real-world cohort study, in which mavacamten was prescribed at the patients’ own expense for all cases, clearly demonstrated this, as approximately 20% of patients discontinued the treatment due to its high cost rather than anticipated medical adverse events [30].

Meanwhile, SRT, especially septal myectomy, is associated with considerable variability in procedural risk depending on institutional and surgeon’s experience [13]. A study showed that in-hospital mortality after septal myectomy tripled in lowest tertile of myectomy volume hospitals [13]. Moreover, accessibility of SRTs is still largely limited in many developing countries. In a Latin American cohort, only 3.9% of patients could undergo septal myectomy [46]. In contrast, pharmacologic therapy with mavacamten offers a non-invasive alternative that may be more easily standardized across diverse practice settings.

### Disease-modifying effect

The advent of CMIs has opened several investigative frontiers in HCM. A key question is whether long-term use of these agents can favorably alter the natural history of HCM beyond symptomatic improvement. Indeed, there is little rationale to use CMI in asymptomatic oHCM, as all studies were conducted on symptomatic patients. Early indicators of reverse remodeling with mavacamten, such as reduced LVMI, LAVI, LV global longitudinal strain, left atrial (LA) strain, and E/e’ values, are intriguing [23, 24, 30, 47, 48]. Ongoing extension studies will be needed to clarify if sustained myosin inhibition leads to regression of hypertrophy and consequently impact on hard outcomes like AF, heart failure hospitalizations, sudden cardiac death or any cardiac mortality; while improvements in LVMI, LV or LA strain, and diastolic function could theoretically lower risk, changes in these values might not predict HCM-related cardiovascular outcomes [49–53]. Furthermore, equally important is the clinical implication of LVEF reduction during treatment. Since lower LVEF is associated with adverse outcomes in HCM [54], whether the modest, but sustained decline in LVEF with CMI may offset the potential benefits of reverse remodeling remains an important area for future investigation. In addition, whether disease-modifying effects are valid in nHCM should also be verified.

### Streamlining monitoring process

The REMS Program for mavacamten ensures safety and should be viewed as analogous to warfarin monitoring or other drugs requiring periodic monitoring. As experience accumulates, these processes may streamline, especially if aficamten proves to require less intensive surveillance. As previously suggested, NT-proBNP may somewhat help simplifying repeated follow-up for stable patients [30]. Artificial intelligence (AI) technology may also help simplifying the monitoring processes. Indeed, AI electrocardiogram has been validated in multiethnic cohorts to diagnose HCM [55]. Recently, the output values of this model were demonstrated to correlate well with longitudinal hemodynamic changes in LVOT pressure gradients as well as serum NT-proBNP levels in oHCM patients on mavacamten [56]. Altogether, serum and electric biomarkers are promising, but the safety of their use should be further validated in the future prospective study. Machine-learning technology is promising beyond monitoring process in HCM, which requires another validation in the CMI management era [57].

### Upcoming trials

The ongoing phase 3 MAPLE-HCM trial is directly comparing aficamten against metoprolol in oHCM, which will provide insights into how CMI stacks up against a β-blocker as first-line therapy [58]. If CMI proves superior in improving exercise capacity and quality of life, it could shift the initial treatment paradigm for oHCM.

In nHCM, the phase 3 ODYSSEY-HCM and ACACIA-HCM trials will be crucial. Although some promising results were demonstrated in the phase 2 studies in nHCM, preliminary results from ODYSSEY-HCM trial showed no benefit of mavacamten in the primary endpoint. Final reports along with subgroup analyses and ACACIA-HCM results are eagerly awaited [32, 34].

### CMI outside HCM

Emerging data also hint at potential use of CMIs outside classical HCM. There is a growing interest in hyperdynamic subtypes of heart failure with preserved ejection fraction (HFpEF): some patients with HFpEF have small hypertrophic ventricles with or without outflow tract obstruction and super-normal systolic function with diastolic dysfunction contributing to increased intracardiac pressures. Indeed, a recent small study on 30 patients with HFpEF with LVEF ≥ 60% has demonstrated that mavacamten use for 26 weeks was associated with significant 26% reduction in NT-proBNP level, 20% reduction in troponin I level, improvement in NYHA functional class in 42% and improvement in diastolic parameters of E/e’ and LAVI [59]. It should be noted that a decrease in LVEF was only modest, showing − 3.2% decrease over 26 weeks and none of the patients discontinued the study drug due to side effects of mavacamten. However, caution is exercised, as the safety and efficacy of reducing contractility in HFpEF remain to be established.

## Conclusions

CMIs have rapidly progressed from novel concept to real-world application in managing oHCM. Both mavacamten and aficamten have shown that directly targeting hypercontractility can alleviate dynamic obstruction, improve exercise capacity, and enhance quality of life for oHCM patients. As these drugs have already become integrated into clinical practice in the management of oHCM, ongoing research needs to address their optimal use; i.e., determining how early to start therapy, how long to continue, how they compare or combine with existing treatments, and most importantly, whether they can change clinical course and hard outcomes of HCM. Upcoming studies will provide critical data on the role of CMIs in long-term disease modification, as well as their roles possibly in nHCM or HFpEF.

## Supplementary Information

Supplementary Material 1.

Supplementary Material 2.

Supplementary Material 3.

Supplementary Material 4.

## Data Availability

No datasets were generated or analysed during the current study.

## Abbreviations

AF: Atrial fibrillation
AI: Artificial intelligence
CMI: Cardiac myosin inhibitor
FDA: Food and Drug Administration
HCM: Hypertrophic cardiomyopathy
HFpEF: Heart failure with preserved ejection fraction
ICD: Implantable cardioverter-defibrillator
KCCQ-CSS: Kansas City Cardiomyopathy Questionnaire–Clinical Symptom Score
LA: Left atrial
LAVI: Left atrial volume index
LV: Left ventricular
LVEF: Left ventricular ejection fraction
LVMI: Left ventricular mass index
LVOT: Left ventricular outflow tract
NT-proBNP: N-terminal pro–B-type natriuretic peptide
NYHA: New York Heart Association
nHCM: Nonobstructive hypertrophic cardiomyopathy
oHCM: Obstructive hypertrophic cardiomyopathy
pVO_2_: Peak oxygen consumption
REMS: Risk Evaluation and Mitigation Strategy
SRT: Septal reduction therapy

## References

[1] Maron BJ, Ommen SR, Semsarian C, Spirito P, Olivotto I, Maron MS, et al. Hypertrophic cardiomyopathy: present and future, with translation into contemporary cardiovascular medicine. J Am Coll Cardiol. 2014;64:83–99. 24998133 10.1016/j.jacc.2014.05.003

[2] Maron BJ, Gardin JM, Flack JM, Gidding SS, Kurosaki TT, Bild DE, et al. Prevalence of hypertrophic cardiomyopathy in a general population of young adults. Echocardiographic analysis of 4111 subjects in the CARDIA Study. Circulation. 1995;92:785–9. 10.1161/01.cir.92.4.785 7641357

[3] Semsarian C, Ingles J, Maron MS, Maron BJ. New perspectives on the prevalence of hypertrophic cardiomyopathy. J Am Coll Cardiol. 2015;65:1249–54. 25814232 10.1016/j.jacc.2015.01.019

[4] Braunwald E, Lambrew CT, Rockoff SD, Ross J, Morrow AG. Idiopathic hypertrophic subaortic stenosis. I. A description of the disease based upon an analysis of 64 patients. Circulation. 1964;30:3–119. 10.1161/01.cir.29.5s4.iv-3 14227306

[5] Olivotto I, Cecchi F, Casey SA, Dolara A, Traverse JH, Maron BJ, et al. Impact of atrial fibrillation on the clinical course of hypertrophic cardiomyopathy. Circulation. 2001;104:2517–24. 11714644 10.1161/hc4601.097997

[6] Maron BJ, Olivotto I, Bellone P, Conte MR, Cecchi F, Flygenring BP, et al. Clinical profile of stroke in 900 patients with hypertrophic cardiomyopathy. J Am Coll Cardiol. 2002;39:301–7. 11788223 10.1016/s0735-1097(01)01727-2

[7] Maron BJ. Hypertrophic cardiomyopathy: a systematic review. JAMA. 2002;287:1308–20. 11886323 10.1001/jama.287.10.1308

[8] Kwak S, Kim J, Park CS, Lee HJ, Park JB, Lee SP, et al. Distinct phenotypic groups and related clinical outcomes in patients with hypertrophic cardiomyopathy. J Am Heart Assoc. 2024;13: e036245. 39392146 10.1161/JAHA.124.036245 PMC11935599

[9] Ommen SR, Ho CY, Asif IM, Balaji S, Burke MA, Day SM, et al. 2024 AHA/ACC/AMSSM/HRS/PACES/SCMR guideline for the management of hypertrophic cardiomyopathy: a report of the American Heart Association/American College of Cardiology Joint Committee on Clinical Practice Guidelines. J Am Coll Cardiol. 2024;83:2324–405. 38727647 10.1016/j.jacc.2024.02.014

[10] Arbelo E, Protonotarios A, Gimeno JR, Arbustini E, Barriales-Villa R, Basso C, et al. 2023 ESC guidelines for the management of cardiomyopathies. Eur Heart J. 2023;44:3503–626. 37622657 10.1093/eurheartj/ehad194

[11] Sherrid MV, Barac I, McKenna WJ, Elliott PM, Dickie S, Chojnowska L, et al. Multicenter study of the efficacy and safety of disopyramide in obstructive hypertrophic cardiomyopathy. J Am Coll Cardiol. 2005;45:1251–8. 15837258 10.1016/j.jacc.2005.01.012

[12] Maron BJ. Clinical course and management of hypertrophic cardiomyopathy. N Engl J Med. 2018;379:655–68. 30110588 10.1056/NEJMra1710575

[13] Kim LK, Swaminathan RV, Looser P, Minutello RM, Wong SC, Bergman G, et al. Hospital volume outcomes after septal myectomy and alcohol septal ablation for treatment of obstructive hypertrophic cardiomyopathy: US nationwide inpatient database, 2003–2011. JAMA Cardiol. 2016;1:324–32. 27438114 10.1001/jamacardio.2016.0252

[14] Lee HJ, Kim J, Chang SA, Kim YJ, Kim HK, Lee SC, et al. Major clinical issues in hypertrophic cardiomyopathy. Korean Circ J. 2022;52:563–75. 35929051 10.4070/kcj.2022.0159 PMC9353251

[15] Keam SJ. Mavacamten: first approval. Drugs. 2022;82:1127–35. 35802255 10.1007/s40265-022-01739-7 PMC9338109

[16] Anderson RL, Trivedi DV, Sarkar SS, Henze M, Ma W, Gong H, et al. Deciphering the super relaxed state of human β-cardiac myosin and the mode of action of mavacamten from myosin molecules to muscle fibers. Proc Natl Acad Sci U S A. 2018;115:E8143–52. 30104387 10.1073/pnas.1809540115 PMC6126717

[17] Olivotto I, Oreziak A, Barriales-Villa R, Abraham TP, Masri A, Garcia-Pavia P, et al. Mavacamten for treatment of symptomatic obstructive hypertrophic cardiomyopathy (EXPLORER-HCM): a randomised, double-blind, placebo-controlled, phase 3 trial. Lancet. 2020;396:759–69. 32871100 10.1016/S0140-6736(20)31792-X

[18] Desai MY, Owens A, Geske JB, Wolski K, Naidu SS, Smedira NG, et al. Myosin inhibition in patients with obstructive hypertrophic cardiomyopathy referred for septal reduction therapy. J Am Coll Cardiol. 2022;80:95–108. 35798455 10.1016/j.jacc.2022.04.048

[19] Nag S, Trivedi DV, Sarkar SS, Adhikari AS, Sunitha MS, Sutton S, et al. The myosin mesa and the basis of hypercontractility caused by hypertrophic cardiomyopathy mutations. Nat Struct Mol Biol. 2017;24:525–33. 28481356 10.1038/nsmb.3408 PMC5737966

[20] Ormerod JO, Frenneaux MP, Sherrid MV. Myocardial energy depletion and dynamic systolic dysfunction in hypertrophic cardiomyopathy. Nat Rev Cardiol. 2016;13:677–87. 27411403 10.1038/nrcardio.2016.98

[21] Green EM, Wakimoto H, Anderson RL, Evanchik MJ, Gorham JM, Harrison BC, et al. A small-molecule inhibitor of sarcomere contractility suppresses hypertrophic cardiomyopathy in mice. Science. 2016;351:617–21. 26912705 10.1126/science.aad3456 PMC4784435

[22] Chuang C, Collibee S, Ashcraft L, Wang W, Vander Wal M, Wang X, et al. Discovery of aficamten (CK-274), a next-generation cardiac myosin inhibitor for the treatment of hypertrophic cardiomyopathy. J Med Chem. 2021;64:14142–52. 34606259 10.1021/acs.jmedchem.1c01290

[23] Saberi S, Cardim N, Yamani M, Schulz-Menger J, Li W, Florea V, et al. Mavacamten favorably impacts cardiac structure in obstructive hypertrophic cardiomyopathy: EXPLORER-HCM cardiac magnetic resonance substudy analysis. Circulation. 2021;143:606–8. 33190524 10.1161/CIRCULATIONAHA.120.052359

[24] Hegde SM, Lester SJ, Solomon SD, Michels M, Elliott PM, Nagueh SF, et al. Effect of mavacamten on echocardiographic features in symptomatic patients with obstructive hypertrophic cardiomyopathy. J Am Coll Cardiol. 2021;78:2518–32. 34915982 10.1016/j.jacc.2021.09.1381

[25] Garcia-Pavia P, Oręziak A, Masri A, Barriales-Villa R, Abraham TP, Owens AT, et al. Long-term effect of mavacamten in obstructive hypertrophic cardiomyopathy. Eur Heart J. 2024;45:5071–83. 39217450 10.1093/eurheartj/ehae579 PMC11646600

[26] Desai MY, Owens A, Geske JB, Wolski K, Saberi S, Wang A, et al. Dose-blinded myosin inhibition in patients with obstructive hypertrophic cardiomyopathy referred for septal reduction therapy: outcomes through 32 weeks. Circulation. 2023;147:850–63. 36335531 10.1161/CIRCULATIONAHA.122.062534

[27] Desai MY, Owens A, Wolski K, Geske JB, Saberi S, Wang A, et al. Mavacamten in patients with hypertrophic cardiomyopathy referred for septal reduction: week 56 results from the VALOR-HCM randomized clinical trial. JAMA Cardiol. 2023;8:968–77. 37639243 10.1001/jamacardio.2023.3342 PMC10463171

[28] Desai MY, Wolski K, Owens A, Geske JB, Saberi S, Wang A, et al. Mavacamten in patients with hypertrophic cardiomyopathy referred for septal reduction: week 128 results from VALOR-HCM. Circulation. 2025;151:1378–90. 39556124 10.1161/CIRCULATIONAHA.124.072445 PMC12063683

[29] Tian Z, Li L, Li X, Wang J, Zhang Q, Li Z, et al. Effect of mavacamten on Chinese patients with symptomatic obstructive hypertrophic cardiomyopathy: the EXPLORER-CN randomized clinical trial. JAMA Cardiol. 2023;8:957–65. 37639259 10.1001/jamacardio.2023.3030 PMC10463173

[30] Lim J, Cho JY, Kwak S, Park CS, Park J, Choi HM, et al. Real-world experience of mavacamten for patients with obstructive hypertrophic cardiomyopathy in South Korea: a prospective multi-center observational study. Korean Circ J. 2025;55:339–54. 40169351 10.4070/kcj.2024.0443 PMC12046305

[31] Kitaoka H, Ieda M, Ebato M, Kozuma K, Takayama M, Tanno K, et al. Phase 3 open-label study evaluating the efficacy and safety of mavacamten in Japanese adults with obstructive hypertrophic cardiomyopathy: the HORIZON-HCM study. Circ J. 2024;89:130–8. 39505542 10.1253/circj.CJ-24-0501

[32] Ho CY, Mealiffe ME, Bach RG, Bhattacharya M, Choudhury L, Edelberg JM, et al. Evaluation of mavacamten in symptomatic patients with nonobstructive hypertrophic cardiomyopathy. J Am Coll Cardiol. 2020;75:2649–60. 32466879 10.1016/j.jacc.2020.03.064

[33] Maron MS, Masri A, Nassif ME, Barriales-Villa R, Arad M, Cardim N, et al. Aficamten for symptomatic obstructive hypertrophic cardiomyopathy. N Engl J Med. 2024;390:1849–61. 38739079 10.1056/NEJMoa2401424

[34] Masri A, Sherrid MV, Abraham TP, Choudhury L, Garcia-Pavia P, Kramer CM, et al. Efficacy and safety of aficamten in symptomatic nonobstructive hypertrophic cardiomyopathy: results from the REDWOOD-HCM trial, cohort 4. J Card Fail. 2024;30:1439–48. 38493832 10.1016/j.cardfail.2024.02.020

[35] Park CS, Rhee TM, Lee HJ, Yoon YE, Park JB, Lee SP, et al. Prognostic and safety implications of renin-angiotensin-aldosterone system inhibitors in hypertrophic cardiomyopathy: a real-world observation over 2,000 patients. Korean Circ J. 2023;53:606–18. 37653696 10.4070/kcj.2023.0035 PMC10475688

[36] Oh IY, Park KW, Kang SH, Park JJ, Na SH, Kang HJ, et al. Association of cytochrome P450 2C19*2 polymorphism with clopidogrel response variability and cardiovascular events in Koreans treated with drug-eluting stents. Heart. 2012;98:139–44. 21700758 10.1136/hrt.2011.227272

[37] Ramsjö M, Aklillu E, Bohman L, Ingelman-Sundberg M, Roh HK, Bertilsson L, et al. CYP2C19 activity comparison between Swedes and Koreans: effect of genotype, sex, oral contraceptive use, and smoking. Eur J Clin Pharmacol. 2010;66:871–7. 20499227 10.1007/s00228-010-0835-0

[38] Tjahjadi C, Butcher SC, Zegkos T, Sia CH, Hirasawa K, Kamperidis V, et al. Differences in characteristics and outcomes between patients with hypertrophic cardiomyopathy from Asian and European centers. J Am Heart Assoc. 2022;11: e023313. 35574964 10.1161/JAHA.121.023313 PMC9238568

[39] Desai MY, Seto D, Cheung M, Afsari S, Patel N, Bastien A, et al. Mavacamten: real-world experience from 22 months of the Risk Evaluation and Mitigation Strategy (REMS) Program. Circ Heart Fail. 2025;18: e012441. 39523955 10.1161/CIRCHEARTFAILURE.124.012441 PMC11745710

[40] McKeown LA. Mavacamten strikes out in phase III trial of nonobstructive HCM [Internet]. TCTMD; 2025 [cited 2025 Apr 23]. Available from: https://www.tctmd.com/news/mavacamten-strikes-out-phase-iii-trial-nonobstructive-hcm

[41] Maron MS, Masri A, Choudhury L, Olivotto I, Saberi S, Wang A, et al. Phase 2 study of aficamten in patients with obstructive hypertrophic cardiomyopathy. J Am Coll Cardiol. 2023;81:34–45. 36599608 10.1016/j.jacc.2022.10.020

[42] Kwon DH, Smedira NG, Rodriguez ER, Tan C, Setser R, Thamilarasan M, et al. Cardiac magnetic resonance detection of myocardial scarring in hypertrophic cardiomyopathy: correlation with histopathology and prevalence of ventricular tachycardia. J Am Coll Cardiol. 2009;54:242–9. 19589437 10.1016/j.jacc.2009.04.026

[43] Siontis KC, Geske JB, Ong K, Nishimura RA, Ommen SR, Gersh BJ, et al. Atrial fibrillation in hypertrophic cardiomyopathy: prevalence, clinical correlations, and mortality in a large high-risk population. J Am Heart Assoc. 2014;3: e001002. 24965028 10.1161/JAHA.114.001002 PMC4309084

[44] Braunwald E, Saberi S, Abraham TP, Elliott PM, Olivotto I. Mavacamten: a first-in-class myosin inhibitor for obstructive hypertrophic cardiomyopathy. Eur Heart J. 2023;44:4622–33. 37804245 10.1093/eurheartj/ehad637 PMC10659958

[45] Malik FI, Robertson LA, Armas DR, Robbie EP, Osmukhina A, Xu D, et al. A phase 1 dose-escalation study of the cardiac myosin inhibitor aficamten in healthy participants. JACC Basic Transl Sci. 2022;7:763–75. 36061336 10.1016/j.jacbts.2022.04.008 PMC9436819

[46] Espinola-Zavaleta N, Vega A, Basto DM, Alcantar-Fernández AC, GuarnerLans V, Soto ME, et al. Survival and clinical behavior of hypertrophic cardiomyopathy in a latin american cohort in contrast to cohorts from the developed world. J Cardiovasc Ultrasound. 2015;23:20–6. 25883752 10.4250/jcu.2015.23.1.20 PMC4398780

[47] Desai MY, Okushi Y, Wolski K, Geske JB, Owens A, Saberi S, et al. Mavacamten-associated temporal changes in left atrial function in obstructive HCM: insights from the VALOR-HCM trial. JACC Cardiovasc Imaging. 2025;18:251–62. 39254622 10.1016/j.jcmg.2024.08.005

[48] Desai MY, Okushi Y, Gaballa A, Wang Q, Geske JB, Owens AT, et al. Serial changes in ventricular strain in symptomatic obstructive hypertrophic cardiomyopathy treated with mavacamten: insights from the VALOR-HCM trial. Circ Cardiovasc Imaging. 2024;17: e017185. 39221824 10.1161/CIRCIMAGING.124.017185 PMC11410149

[49] Lee HJ, Kim HK, Lee SC, Kim J, Park JB, Hwang IC, et al. Supplementary role of left ventricular global longitudinal strain for predicting sudden cardiac death in hypertrophic cardiomyopathy. Eur Heart J Cardiovasc Imaging. 2022;23:1108–16. 34542591 10.1093/ehjci/jeab187

[50] Lee HJ, Kim HK, Rhee TM, Choi YJ, Hwang IC, Yoon YE, et al. Left atrial reservoir strain-based left ventricular diastolic function grading and incident heart failure in hypertrophic cardiomyopathy. Circ Cardiovasc Imaging. 2022;15: e013556. 35439039 10.1161/CIRCIMAGING.121.013556

[51] Kim S, Chung WJ. Longitudinal changes of left atrial volume index as a prognosticator in hypertrophic cardiomyopathy. J Cardiovasc Imaging. 2023;31:96–7. 37096674 10.4250/jcvi.2022.0143 PMC10133809

[52] Kim K, Lee SD, Lee HJ, Kim H, Kim HR, Cho YH, et al. Role and clinical importance of progressive changes in echocardiographic parameters in predicting outcomes in patients with hypertrophic cardiomyopathy. J Cardiovasc Imaging. 2023;31:85–95. 37096673 10.4250/jcvi.2022.0053 PMC10133807

[53] Kwak S, Kim J, Park CS, Lee HJ, Park JB, Lee SP, et al. Prognostic implication of left ventricular global longitudinal strain in patients with hypertrophic cardiomyopathy and coexisting hypertension. Korean Circ J. 2025; 10.4070/kcj.2024.0213 PMC12270826 39962967

[54] Choi YJ, Kim HK, Hwang IC, Park CS, Rhee TM, Lee HJ, et al. Prognosis of patients with hypertrophic cardiomyopathy and low-normal left ventricular ejection fraction. Heart. 2023;109:771–8. 36581445 10.1136/heartjnl-2022-321853

[55] Siontis KC, Wieczorek MA, Maanja M, Hodge DO, Kim HK, Lee HJ, et al. Hypertrophic cardiomyopathy detection with artificial intelligence electrocardiography in international cohorts: an external validation study. Eur Heart J Digit Health. 2024;5:416–26. 39081936 10.1093/ehjdh/ztae029 PMC11284003

[56] Siontis KC, Abreau S, Attia ZI, Barrios JP, Dewland TA, Agarwal P, et al. Patient-level artificial intelligence-enhanced electrocardiography in hypertrophic cardiomyopathy: longitudinal treatment and clinical biomarker correlations. JACC Adv. 2023;2: 100582. 38076758 10.1016/j.jacadv.2023.100582 PMC10702858

[57] Rhee TM, Ko YK, Kim HK, Lee SB, Kim BS, Choi HM, et al. Machine learning-based discrimination of cardiovascular outcomes in patients with hypertrophic cardiomyopathy. JACC Asia. 2024;4:375–86. 38765660 10.1016/j.jacasi.2023.12.001 PMC11099823

[58] Garcia-Pavia P, Bilen O, Burroughs M, Costabel JP, de Barros Correia E, Dybro AM, et al. Aficamten vs. metoprolol for obstructive hypertrophic cardiomyopathy: MAPLE-HCM rationale, study design, and baseline characteristics. JACC Heart Fail. 2025;13:346–57. 10.1016/j.jchf.2024.11.011 39909646

[59] Shah SJ, Rigolli M, Javidialsaadi A, Patel RB, Khadra S, Goyal P, et al. Cardiac myosin inhibition in heart failure with normal and supranormal ejection fraction: primary results of the EMBARK-HFpEF trial. JAMA Cardiol. 2025;10:170–5. 39347697 10.1001/jamacardio.2024.3810 PMC11822545

